# Combined Use of the Canine Adenovirus-2 and DREADD-Technology to Activate Specific Neural Pathways *In Vivo*

**DOI:** 10.1371/journal.pone.0095392

**Published:** 2014-04-15

**Authors:** Arjen J. Boender, Johannes W. de Jong, Linde Boekhoudt, Mieneke C. M. Luijendijk, Geoffrey van der Plasse, Roger A. H. Adan

**Affiliations:** Department of Translational Neuroscience, Brain Center Rudolf Magnus, University Medical Center Utrecht, Utrecht, The Netherlands; Roma Tre University, Italy

## Abstract

We here describe a technique to transiently activate specific neural pathways *in vivo*. It comprises the combined use of a CRE-recombinase expressing canine adenovirus-2 (CAV-2) and an adeno-associated virus (AAV-hSyn-DIO-hM_3_D(G_q_)-mCherry) that contains the floxed inverted sequence of the designer receptor exclusively activated by designer drugs (DREADD) hM_3_D(G_q_)-mCherry. CAV-2 retrogradely infects projection neurons, which allowed us to specifically express hM_3_D(G_q_)-mCherry in neurons that project from the ventral tegmental area (VTA) to the nucleus accumbens (Acb), the majority of which were dopaminergic. Activation of hM_3_D(G_q_)-mCherry by intraperitoneal (i.p.) injections of clozapine-N-oxide (CNO) leads to increases in neuronal activity, which enabled us to specifically activate VTA to Acb projection neurons. The VTA to Acb pathway is part of the mesolimbic dopamine system and has been implicated in behavioral activation and the exertion of effort. Injections of all doses of CNO led to increases in progressive ratio (PR) performance. The effect of the lowest dose of CNO was suppressed by administration of a DRD1-antagonist, suggesting that CNO-induced increases in PR-performance are at least in part mediated by DRD1-signaling. We hereby validate the combined use of CAV-2 and DREADD-technology to activate specific neural pathways and determine consequent changes in behaviorally relevant paradigms.

## Introduction

While the existence of neuronal pathways has been appreciated since Ramon y Cajal’s time, techniques to specifically activate them *in vivo* and study consequent changes in behavior have only been developed recently. The combination of optogenetic and designer receptor exclusively activated by designer drugs (DREADD) technologies with CRE-mediated homologous recombination enables specific targeting and manipulation of neural pathways and has improved our understanding of how the activity of specific neuronal pathways can act as determinants of behavior [1], [2]. Most often however, optogenetic and DREADD technologies are combined with mouse CRE-lines, which limit their applicability. As suggested by Nair et al. [3], the combination of these technologies with CRE-expressing viral vectors provides another means to target specific neural pathways on a cellular resolution. Here, we test and validate the combined use of CAV-2 and DREADD-technology to pharmacogenetically activate specific neural pathways *in vivo*.

The technique entails the infusion of two viral vectors (CAV-2 and AAV-hSyn-DIO-hM_3_D(G_q_)-mCherry) into two sites that are connected through direct neuronal projections and represent a neuronal pathway. AAV-hSyn-DIO-hM_3_D(G_q_)-mCherry is infused in the site where the cell bodies are located, while CAV-2 is infused in the area that is innervated by the corresponding axons. After infection of axonal terminals, CAV-2 is transported towards the cell bodies and expresses CRE-recombinase [4], [5]. AAV-hSyn-DIO-hM_3_D(G_q_)-mCherry contains the floxed inverted sequence of hM_3_D(G_q_)-mCherry, which is reoriented in the presence of CRE, prompting the expression of hM_3_D(G_q_)-mCherry. This ensures that hM_3_D(G_q_)-mCherry is not expressed in all AAV-hSyn-DIO-hM_3_D(G_q_)-mCherry infected neurons, but exclusively in those that are also infected with CAV-2.

HM_3_D(G_q_)-mCherry is a G-protein coupled receptor that is activated by the pharmacologically inert compound clozapine-N-oxide CNO [6]. The binding of CNO to hM_3_D(G_q_)-mCherry initiates several intracellular cascades [7], which leads to increases in neuronal activity [1], [8]. CNO crosses the blood-brain barrier and can be administered i.p., which facilitates its use in behavioral experiments. As CNO only activates those neurons in which hM_3_D(G_q_)-mCherry is expressed, the combination of hM_3_D(G_q_)-mCherry with CAV-2 allows for the activation of specific neural pathways.

To validate the combined use of CAV-2 and DREADD-technology to activate a specific neural pathway and investigate consequent changes in behavior, we chose to activate a neural pathway of which the function is relatively well-described: the VTA to Acb pathway. Activity of this pathway has been suggested to increase locomotor activity and enhance performance rates in several tasks that demand effort (for reviews see: Salomone et al. 2007 and Meredith et al. 2008). Its effect on behavioral activation is believed to primarily constitute dopaminergic neurotransmission, in particular via dopamine receptor D1 (DRD1) signaling [9], [10]. Indeed, we find that the majority of transduced neurons in this pathway are dopaminergic and that its activation does lead to increases in locomotor activity and enhances performance rates in a PR-paradigm. In a subsequent experiment, we show that the effect of the lowest dose of CNO on PR-performance can be suppressed by administration of the DRD1-antagonist SCH23390, thereby underscoring the potential of the combined use of CAV-2 and DREADD-technology to provide means to bridge the gap that separates structure from function.

## Materials and Methods

### Ethics Statement

Experiments were approved by the Animal Ethics Committee of the university of Utrecht and were conducted in agreement with Dutch laws (Wet op de Dierproeven, 1996) and European regulations (Guideline 86/609/EEC).

### Animals

Adult male Wistar rats (Charles-River, Germany) were used (*n = *15). The rats were housed individually (378×217×180 cm) in a controlled environment under a reversed 12∶12 light/dark cycle, with lights on at 1900 h. In their home cage, rats had *ad lib* access to standard chow (Special Diet Service, UK) and water.

### Operant Conditioning

Experiments were conducted in four rat operant conditioning chambers (30.5×24.2×21.0 cm; Med Associates, USA) placed within sound attenuated and ventilated boxes. The operant boxes were equipped with two cue-lights, a pellet-dispenser, a receptacle for 45 mg sucrose pellets (SP; 5TUL, TestDiet, USA) and two retractable levers. The cue lights were located above the retractable levers and the receptacle was placed in the middle. Training of the rats was performed between 1100 h and 1600 h in a fixed ratio 1 paradigm (FR1) with a total duration of 0.5 h and a maximum of 60 trials. During each trial both levers were present, but only presses on the active lever (ALP) led to deliverance of SP. During the 20 s inter trial interval that followed SP delivery, the levers were retracted and the cue-light above the active lever was activated, after which a new trial started and levers were presented to the animal again. Pressing the inactive lever (ILP) did not lead to deliverance of SP, activation of the cue-lights or retraction of the levers. FR1 sessions took place twice a day (at least 2 h apart) for a period of five days, after which all rats reached the training criterion and were considered trained (i.e. three consecutive days >30 obtained SP). Subsequently, the progressive ratio (PR) paradigm was implemented. All PR-sessions started before 1300 h and were completed by 1600 h. The PR-sessions were not restricted in the maximum of number of trials or in time *per se*. However, if SP were not obtained within a 0.5 h period, the PR-session ended. In the PR-paradigm, the number of ALP required to obtain SP is increased with each completed trial (ALP = 5×e^0.2SP^). Successive SP required more ALP, so the amount of ALP reflected the effort that was invested in the task. After five consecutive days of PR-sessions, all rats achieved the training criterion (i.e. three consecutive days >9 obtained SP).

### Surgery

The first group of animals consisted of 7 rats of which 4 rats were bilaterally injected with 1 µl of 1.0×10^∧9^ genomic copies/µl of CAV-2 (IGMM, France) and 3 rats with 1 µl of 5.0×10^∧8^ genomic copies/µl of CAV-2 in the Acb (from bregma: anterioposterior: +1.2 mm, medio-lateral: ±2.8 mm; dorso-ventral: −7,5 mm, at an angle of 10°), using a stereotactic apparatus. Next, all rats were bilaterally injected with 1 µl of 1.0×10^∧9^ genomic copies/µl of AAV-hSyn-DIO-hM_3_D(G_q_)-mCherry (UNC Vector Core, USA) in the VTA (from bregma: anterio-posterior: −5.4 mm, medio-lateral: ±2.2 mm, dorso-ventral: −8.9 mm, at an angle of 10°). As there was no difference observable in VTA hM_3_D(G_q_)-mCherry expression or behavioral measures between the CAV-2 titers, the groups were considered to be equal and combined for statistical analyses. Infusions were performed under fentanyl/fluanisone (0.315 mg/kg fentanyl, 10 mg/kg fluanisone, i.m., Hypnorm, Janssen Pharmaceutica, Belgium) and midazolam (2.5 mg/kg, i.p., Actavis, the Netherlands) anesthesia. Xylocaine was sprayed on the skull to provide local anesthesia (Lidocaine 100 mg/ml, AstraZeneca BV, the Netherlands). A transmitter for the recording of locomotor activity (TA10TA-F40, Data Science International, USA) was placed in the abdominal cavity as well. All rats received three daily peri-surgical injections of carprofen (5 mg/kg, s.c., Carporal, AST Farma BV, the Netherlands) starting at the day of surgery.

A second group of 8 rats underwent identical surgery procedures but received additional guide cannulae (24 gauge; Cooper’s Needleworks, UK) in the Acb (from bregma: anterioposterior: +1.2 mm, medio-lateral: ±2.8 mm; dorso-ventral: −6,5 mm, at an angle of 10°) as well. Cannulae were secured on the skull with stainless steel screw and dental acrylic and stainless steel stylets (29 gauge) were inserted in the guide cannulae to ensure patency.

### Effects of CNO on Operant Conditioning

PR-sessions were initiated 15 d after surgery to allow the CAV-2 to infect the VTA and induce hM_3_D(G_q_)-mCherry expression. The PR-sessions were conducted for a period of six weeks on a daily basis. The standard procedure involved i.p. administration of saline 0.5 h before the start of each PR-session. However, rats were administered CNO (0.1, 0.3 or 1.0 mg/kg, i.p.) when they obtained a stable number of SP (not more than one obtained sucrose pellet deviation between sessions for three consecutive days). The effects of different doses of CNO on the number of ALP and ILP were determined by comparing them to the effect of saline injections the day before and after CNO injection. Statistical analyses were done by conduction of Friedman tests and the Wilcoxon signed rank tests in SPSS 20 (IBM, USA) with the levels of significance set at α = 0.05.

### Effect of CNO on Locomotor Activity

The home cage was placed on a receiver plate (DSI, USA) that received radiofrequency signals from the abdominal transmitter. The plate was connected to software (DSI, USA) that recorded locomotor activity. The summed amount of activity within a period of 4 h was determined, which started 2.5 h after i.p. injection as it was not possible to measure locomotor activity in the operant conditioning chambers. The effects of the different doses of CNO were determined by comparing them to the effects of saline injections the day before and after CNO injection. Statistical analysis were done by conduction of Friedman tests and the Wilcoxon signed rank tests in SPSS 20 (IBM, USA) with the level of significance set at α = 0.05.

### Effects of SCH23390 and CNO on Operant Conditioning

PR-sessions were performed in periods of five days. On days 1 to 3 rats received i.p. injections of saline 0.5 h before the start of PR-sessions, while on days 4 and 5 rats were administered CNO (0.03 or 0.1 mg/kg, i.p.). On day 2 to 5, additional bilateral intra-Acb infusions were performed, either with saline (0.3 µl) or SCH23390 (1.0 µg/0.3 µl; Sigma-Aldrich Co., USA). 5 min. after i.p. injections, 30 gauge injection needles (Bilaney, Germany) were inserted in the guide cannulae and connected to 10 µl Hamilton micro-syringes by polyethylene tubing. Over 60 s, 0.3 µl was infused using a syringe pump (Harvard Apparatus, USA). The injection needles remained inserted for 60 s following infusion, after which stylets were replaced. As described above, effects of different doses of i.p. CNO injections on the number of ALP and ILP were determined by comparing them to the effect of saline injections, but only for saline intra-Acb infusions. The effects of SCH23390 on PR- performance were determined by comparing the effects of intra-Acb infusions of SCH23390 with saline infusions, for each CNO treatment (0, 0.03 and 0.1 mg/kg) separately. In addition, baseline levels (saline i.p. and saline intra-Acb) were compared with the effect of SCH23390 on CNO-induced behaviors (CNO i.p. and SCH23390 intra-Acb). Statistical analyses were done by conduction of Wilcoxon tests in SPSS 20 (IBM, USA) with the levels of significance set at α = 0.05.

### Tissue Preparation

Rats received an overdose of sodium pentobarbital (200 mg/ml, Euthanimal, Alfasan BV, The Netherlands) and were transcardially perfused with 0.1 M phosphate buffered saline (PBS; pH 7.4), followed by PBS containing 4% paraformaldehyde. Brains were excised and kept at 4°C in PBS containing 4% paraformaldehyde for 24 h, transferred to 30% sucrose in PBS and kept at 4°C for 72 h. Brains were snap frozen in isopentane between −60°C and −40°C. Frozen brains were sliced into 40 µm sections using a crysostat (Leica, Germany), collected in PBS containing 25% polyethylene glycol and 25% glycerol and stored at −20°C. Apart from their use in the immunohistochemistry procedures (see below), these sections were also visually inspected to confirm guide cannulae placement in the Acb using a Zeiss Axioskop 2 microscope (Zeiss, Germany).

### Immunohistochemistry

Sections were washed in PBS and subsequently blocked and permeabilized in PBS containing 10% normal goat serum and 0.05% Tween-20 for 1 h. After washing in PBS, sections were incubated overnight with primary antibodies in PBS containing 0.05% Tween-20. HM_3_D(G_q_)-mCherry was detected with mouse anti-mCherry (1∶500, Abcam, UK) and TH was detected with rabbit anti-TH (1∶750, Millipore, USA). After washing in PBS, sections were incubated with secondary antibodies for 1 h. HM_3_D(G_q_)-mCherry was visualized with Alexa-594 labelled goat anti-mouse IgG (Molecular Probes, USA, 1∶500), while TH was identified with Alexa-488 labelled goat anti-rabbit IgG (Molecular Probes, USA, 1∶500). After washing in PBS, sections were washed in PBS and mounted in mounting medium (Calbiochem, USA).

### Image Analysis

Immunofluorescent sections were photographed and digitized using a Zeiss Axioskop 2 epifluorescent microscope (Zeiss, Germany) and a Olympus Fluoview FV1000 confocal microscope (Olympus, Japan). The locus of hM_3_D(G_q_)-mCherry expression was determined by the presence of TH immunoreactivity, which allowed for the identification of the VTA and the SN. In addition, we determined the percentage of hM_3_D(G_q_)-mCherry expressing cells also expressing TH in the first set of animals, using at least one confocal image per animal.

## Results

### Specific Expression of hM_3_D(G_q_)-mCherry in VTA to Acb Projection Neurons

**Figure 1:**
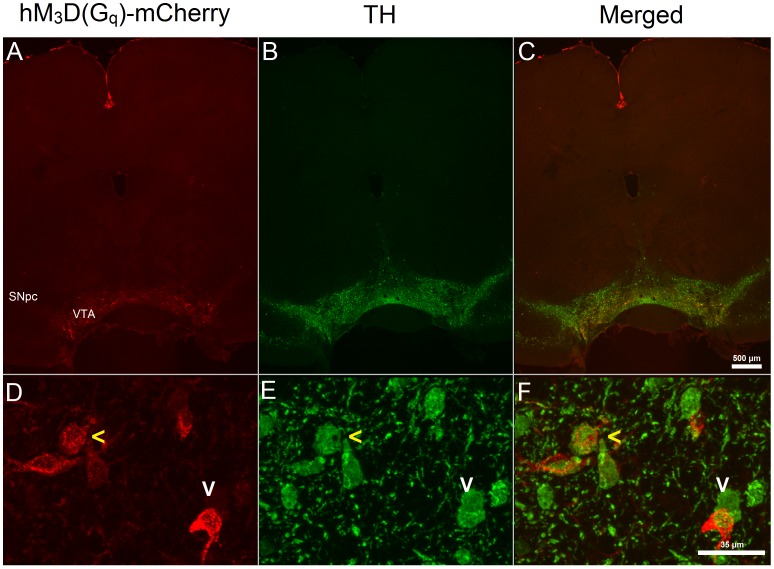
hM_3_D(G_q_)-mCherry is expressed on VTA to Acb neurons. Depicted in the upper row are single section images of (A) hM_3_D(G_q_)-mCherry expression, (B) Tyrosine hydroxylase (TH) expression and (C) combined hM_3_D(G_q_)-mCherry and TH expression. hM_3_D(G_q_)-mCherry expression is restricted to the ventral tegmental area (VTA), as no mCherry expression could be observed in the substantia nigra pars compacta (SNpc) or surrounding areas. Depicted in the lower are single section confocal images of (D) hM_3_D(G_q_)-mCherry expression, (E) TH expression and (F) combined expression of hM_3_D(G_q_)-mCherry and TH. Two populations of hM_3_D(G_q_)-mCherry expressing VTA to nucleus accumbens (Acb) projection neurons can be distinguished, one population that does not express TH (vertical white arrows) and one population that does express TH (horizontal yellow arrows).

Infusions of CAV-2 in the Acb led to expression of CRE in VTA, as hM_3_D(G_q_)-mCherry expression was restricted to this area (figure 1A–C), indicating that CAV-2 specifically infected VTA to Acb projection neurons and expressed CRE, inducing hM_3_D(G_q_)-mCherry expression in the VTA only. One animal was excluded from the group analysis, as no expression of hM_3_D(G_q_)-mCherry was observed, most likely due to a misplaced injection. Two populations of hM_3_D(G_q_)-mCherry positive neurons could be identified (figure 1D-F): one that expressed TH and one that did not, where out of the 91 identified hM_3_D(G_q_)-mCherry positive neurons, 69 did express TH (mean percentage: 78.71±6.09%, data not shown).

### Activation of the VTA to Acb Pathway Leads to Increases in PR-performance as Well as Increased Locomotor Activity

**Figure 2:**
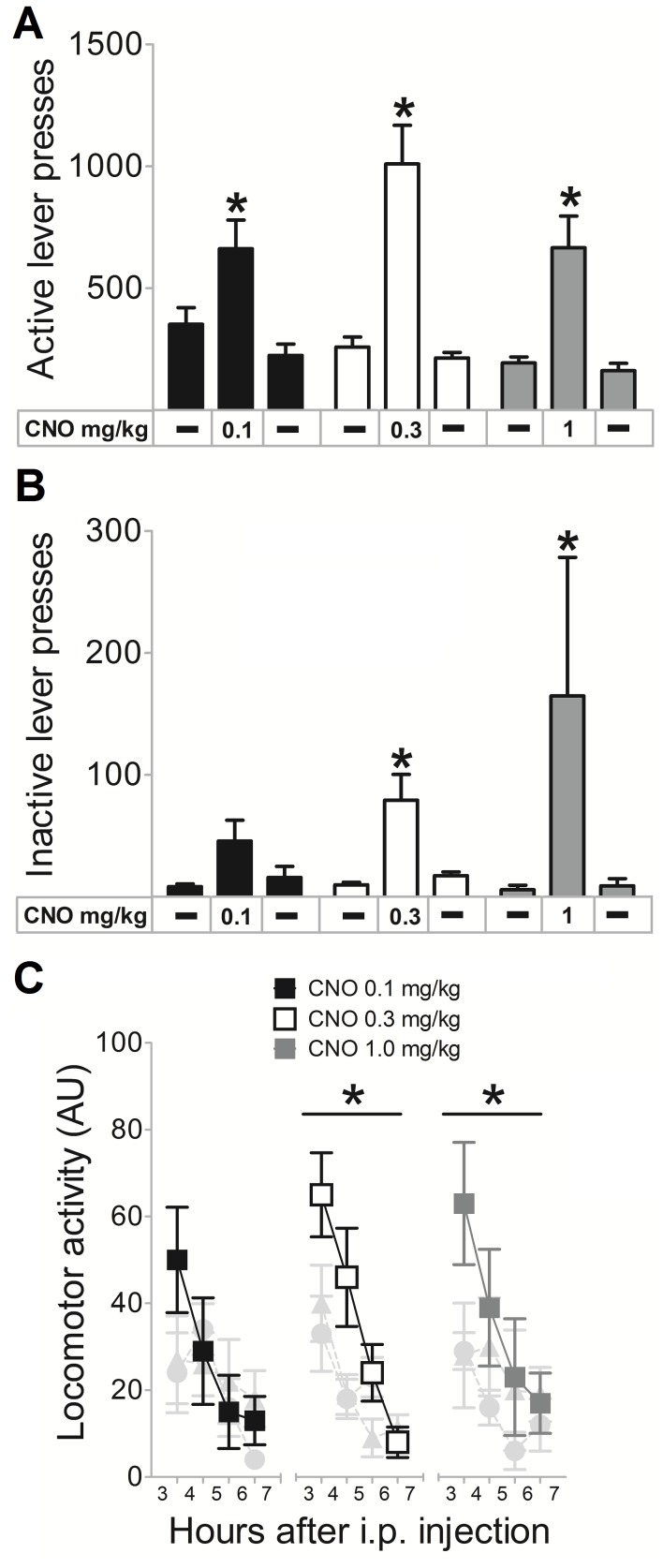
CNO increases PR-performance and locomotor activity. (A) Injections of different doses of clozapine-N-oxide (CNO) led to transient increases in the number of active lever presses. (B) The number of inactive lever presses was dose-dependently affected by injections of CNO. The amount of lever presses are depicted as bars representing group means+SEM. *indicates a significant difference in lever presses between CNO and saline treatments of p<0.05. (C) Injections of CNO also had a transient and dose-dependent effect on locomotor activity. Data in C are presented as the group means of home cage activity ± SEM, binned into one hour periods. Shaded in light gray are circles and triangles that represent the effect of saline injections the day before and after CNO injection respectively. *indicates a significant difference between CNO and saline treatments in locomotor activity 3–7 hours after injections of p<0.05.

Injections of CNO increased the number of ALP (0.1 mg/kg: χ^2^(2) = 10, p = 0.001; 0.3 mg/kg: χ^2^(2) = 18.426, p<0.000; 1 mg/kg χ^2^(2) = 6, p = 0.022). When compared to the first saline injection, all three doses of CNO led to significant increases in the number of ALP (0.1 mg/kg: z = −2.023, p = 0.043; 0.3 mg/kg: z = −3.059, p = 0.002; 1 mg/kg: z = −2.023, p = 0.043). These effects were transient, as the number of ALP returned to baseline the next day when saline was administered (0.1 mg/kg: z = −2.023, p = 0.043; 0.3 mg/kg: z = −3.059, p = 0.002; 1 mg/kg: z = −2.023, p = 0.043) (figure 2A). In the animal in which no hM_3_D(G_q_)-mCherry expression was observed, none of the CNO injections led to increases in the number of ALP (data not shown).

Increases in the number of ILP were dependent on the dose of CNO that was administered (0.1 mg/kg: χ^2^(2) = 5.852, p = 0.054; 0.3 mg/kg: χ^2^(2) = 16.167, p<0.000; 1 mg/kg: χ^2^(2) = 5.333, p = 0.032) (figure 2B). The highest doses of CNO increased the number of ILP significantly (0.3 mg/kg: z = −3.059, p = 0.002; 1 mg/kg: z = −1.993, p = 0.046) and transiently, as the number of ILP returned to baseline after saline administration the next day (0.3 mg/kg: z = −2.886, p = 0.004; 1 mg/kg: z = −0.028, p = 0.028). In the animal in which no hM_3_D(G_q_)-mCherry expression was observed, none of the CNO injections led to increases in the number of ILP (data not shown). Next to increases in the exertion of effort, injections of CNO led to behavioral activation. CNO dose-dependently increased locomotor activity (0.1 mg/kg: χ^2^(2) = 2.8, p = 0.247; 0.3 mg/kg: χ^2^(2) = 9.25, p = 0.015; 1 mg/kg: χ^2^(2) = 8.4, p = 0.015), as only the higher doses of CNO significantly increased locomotor activity, when compared to the saline injection administered the day before (0.3 mg/kg: z = −2.521, p = 0.012; 1 mg/kg: z = −2.023, p = 0.043) (figure 2C). This effect was transient, because when saline was administered the next day, locomotor activity decreased again (0.3 mg/kg: z = −2.380, p = 0.017; 1 mg/kg: z = −2.023, p = 0.043). In the animal in which no hM_3_D(G_q_)-mCherry expression was observed, none of the CNO injections led to increases in locomotor activity (data not shown).

### CNO-induced Increases in PR-performance can be Blocked by Intra-Acb Infusion of the DRD1-antagonist SCH23390

**Figure 3:**
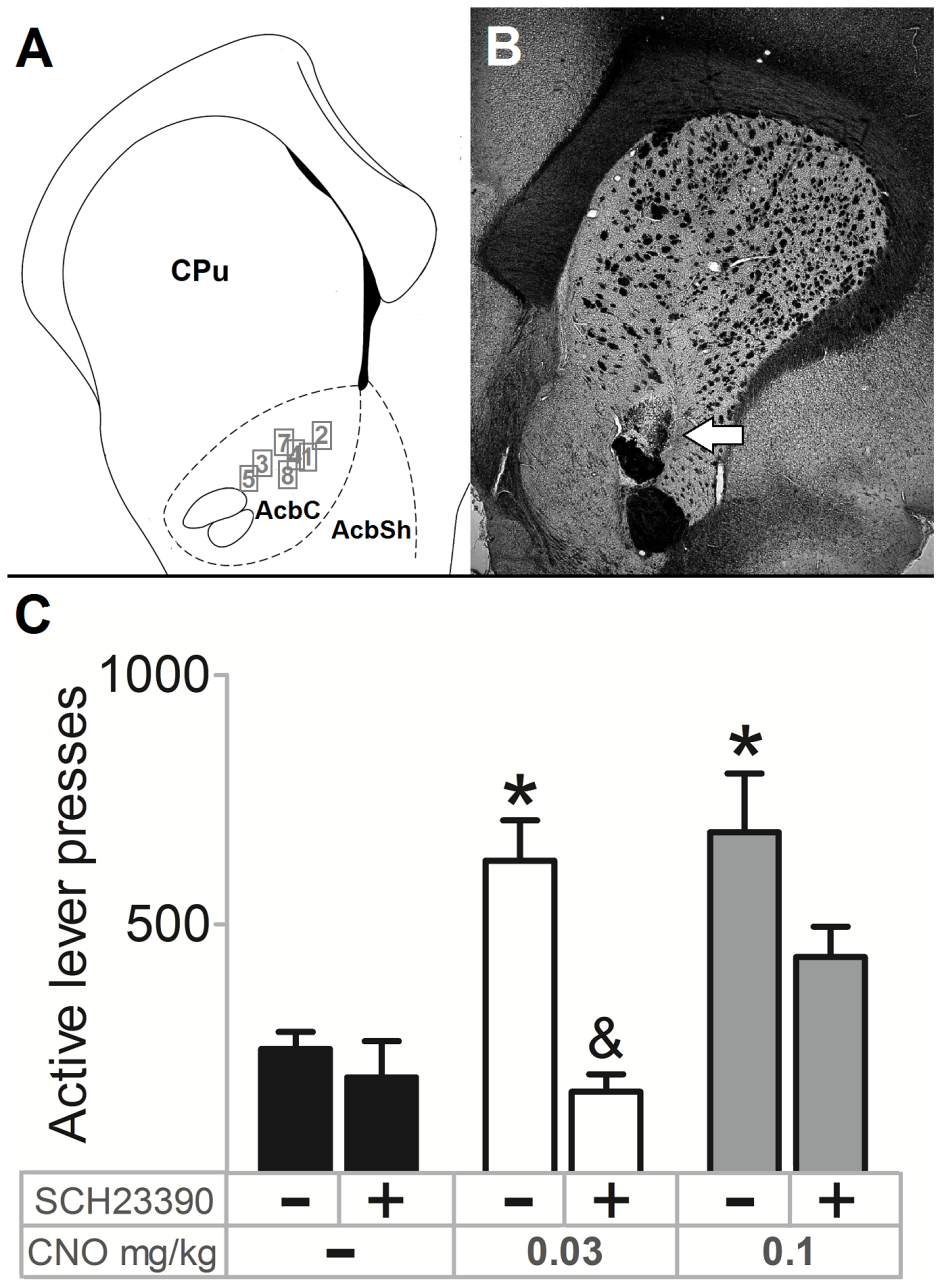
The effect of CNO on PR-performance is dose-dependently suppressed by a DRD1-antagonist. (A) In seven animals cannulae were correctly placed in the nucleus accumbens (Acb). The outlined numbers represent the location of the ending of the guide cannulae, for each animal (1–8) individually. Abbreviations: AcbC = nucleus accumbens core, AcbSh = nucleus accumbens shell, CPu = caudate putamen. (B) Representative image (animal 5) of cannula placement in the Acb. The horizontal arrow indicates the ending of the cannula (C) Intra-Acb infusions of the D1-antagonist SCH22390 dose-dependently suppressed CNO-induced increases in the number of active lever presses. Data are depicted with bars representing group means+SEM, with * indicating a significant difference of i.p. CNO vs. i.p. saline of p<0.05 and & indicating a significant difference of intra-Acb SCH23390 vs. intra-Acb saline of p<0.05.

In one animal, the cannulae were not placed in the Acb, which led to its exclusion from further analyses (figure 3A and B). As before, i.p. CNO injections led to significant increases in the number of ALP (0.03 mg/kg: z = −2.366, p = 0.018; 0.1 mg/kg: z = −2.197, p = 0.028) (figure 3C). Intra-Acb infusion of SCH23390 did not affect the number of ALP when co-administered with saline i.p. (z = −0.847, p = 0.397). As intra-Acb infusion of SCH23390 did not suppress the effect of 0.1 mg/kg CNO on the number of ALP (z = −1.859, p = 0.063) to baseline levels (z = −2.197, p = 0.028), but did show a trend towards suppression, we decided to lower the dose of CNO to 0.03 mg/kg. Now, SCH23390 was able to suppress the effect of CNO on the number of ALP (z = −2.336, p = 0.018) to baseline levels (z = −1.690, p = 0.091).

## Discussion

The combined use of CAV-2 and DREADD-technology allowed us to specifically activate the VTA to Acb pathway and to induce transient increases in PR-performance and locomotor activity. In addition, we provide evidence that the CNO-induced increases in PR-performance are at least in part dependent on DRD1-signaling, as intra-Acb infusion of the DRD1-antagonist SCH23390 suppressed the effect of 0.03 mg/kg CNO. Interestingly, SCH23390 did not suppress the effect of 0.1 mg/kg CNO on PR-performance. Together with the observation that only the highest doses of CNO significantly increased locomotor activity, this suggests a dose-dependent effect of CNO on the activation of hM_3_D(G_q_)-mCherry. In sum, we hereby validate the combined use of CAV-2 and DREADD-technology to activate a specific neural pathway and determine consequent effects on behavior.

This technique has two main advantages over other techniques that can be used to activate specific neural pathways *in vivo*. Firstly, it eliminates the need for cortically implanted glass fibers for the optogenetic manipulation of neural pathways. Secondly, it abolishes the use of transgenic CRE-lines to target specific neural projections on a cellular resolution. Anatomical resolution is achieved by the infusion of two viral vectors in anatomically distinct sites and cellular resolution is warranted by the restricted expression of hM_3_D(G_q_)-mCherry expression in CAV-2 infected neurons, offering means to pharmacologically target specific cellular populations. In principle, this technique could be adapted to genetically target cellular populations within the CAV-2 infected population, by replacing the constitutive hSyn promoter of hM_3_D(G_q_)-mCherry with a cell-specific promoter [11], [12], [13], [14]. If employed in this way, the technique enables the dissociation of the behavioral effects of the different cellular subpopulations within a specific neural pathway. In addition, this technique is easily used for the identification of different cell populations, as we have shown by double immunofluorescent labeling for hM_3_D(G_q_)-mCherry and TH. In sum, the combined use of CAV-2 and DREADD-technology permits the identification of different cellular populations within a specific neural pathway and offers cellular resolution to the behavioral effects that follow its activation, thereby providing a simple tool to pharmacogenetically manipulate the activity of specific neural pathways *in vivo*.
